# Community youth teams facilitating participatory adolescent groups, youth leadership activities and livelihood promotion to improve school attendance, dietary diversity and mental health among adolescent girls in rural eastern India: protocol for a cluster-randomised controlled trial

**DOI:** 10.1186/s13063-019-3984-1

**Published:** 2020-01-08

**Authors:** Suchitra Rath, Audrey Prost, Subhashree Samal, Hemanta Pradhan, Andrew Copas, Sumitra Gagrai, Shibanand Rath, Raj Kumar Gope, Nirmala Nair, Prasanta Tripathy, Komal Bhatia, Kelly Rose-Clarke

**Affiliations:** 1 Ekjut, Chakradharpur, Jharkhand India; 2 Institute for Global Health, University College London, London, UK; 3 Department of Global Health and Social Medicine, King’s College London, Bush House NE Wing, London, WC2B 4BG UK

**Keywords:** Adolescent, Nutrition, Mental health, Education, Participation, Participatory Learning and Action, Peer-led, Youth leadership, Livelihood promotion

## Abstract

**Background:**

Improving the health and development of adolescents aged 10–19 years is a global health priority. One in five adolescents globally live in India. The *Rashtriya Kishor Swasthya Karyakram* (RKSK), India’s national adolescent health strategy, recommends supporting community-based peer educators to conduct group meetings with boys and girls. Groups aim to give adolescents a space to discuss the social and health issues affecting them and build their capacity to become active community members and leaders. There have been no evaluations of the community component of RKSK to date. In this protocol, we describe the evaluation of the Jharkhand Initiative for Adolescent Health (JIAH), a community intervention aligned with RKSK and designed to improve school attendance, dietary diversity and mental health among adolescent girls aged 10–19 years in rural Jharkhand, eastern India.

**Methods:**

The JIAH intervention is delivered by a community youth team consisting of *yuva saathis* (friends of youth), youth leadership facilitators and livelihood promoters. Teams conduct (a) peer-led Participatory Learning and Action meetings with girls and boys, mobilising adolescents, parents, health workers, teachers and the wider community to make changes for adolescent health and development; (b) group-based youth leadership activities to build adolescents’ confidence and resilience; and (c) livelihood promotion with adolescents and their families to provide training and practical skills. We are evaluating the JIAH intervention through a parallel-group, two-arm, superiority, cluster-randomised controlled trial. The unit of randomisation is a geographic cluster of ~1000 people. A total of 38 clusters covering an estimated population of 40,676 have been randomised to control or intervention arms. Nineteen intervention clusters have adolescent groups, youth leadership activities and livelihood promotion. Nineteen control clusters receive livelihood promotion only. Study participants are adolescent girls aged 10–19 years, married or unmarried, in or out of school, living in the study area. Intervention activities are open to all adolescent boys and girls, regardless of their participation in surveys. We will collect data through baseline and endline surveys. Primary trial outcomes are school attendance, dietary diversity and internalising and externalising mental health problems. Secondary outcomes include access to school-related entitlements, emotional or physical violence, self-efficacy and resilience.

**Trial registration:**

ISRCTN17206016. Registered on 27 June 2018.

## Background

### The case for investing in adolescent health globally and in India

Investing in adolescents aged 10–19 years is crucial for their long-term health and well-being and could reduce the inter-generational transmission of undernutrition, violence and poverty [1]. Girls living in low- and middle-income countries (LMICs) are particularly vulnerable due to systematic socio-economic and health disadvantages [2].

Twenty percent of the world’s adolescents live in India [3], where gender inequalities persist across many health and development indicators. One-fourth of girls compared with 8% of boys aged 15–24 have never been to school [4]. In addition, more than half of girls are anaemic, and 45% are underweight [5]. An estimated 7% of girls aged 13–17 have a mental disorder [6]. Improving the psychosocial well-being of adolescent girls in India is thought to be a key strategy to improve their mental and physical health [7]. There is increased recognition of the need for holistic community approaches to adolescent health that cut across multiple sectors, including education, health, nutrition, and protection from violence. In addition, there is growing appreciation that engaging with both girls *and* boys to loosen the hegemonic gender norms that constrain many adolescents is key to achieving long-term gender and health equity [8].

### Evidence gaps

The Indian government’s 2014 adolescent health strategy, the *Rashtriya Kishor Swasthya Karyakram* (RKSK), takes a holistic approach to adolescent health promotion [8]. It comprises facility- and community-based activities with a focus on adolescent participation and leadership. The community component involves adolescent groups facilitated by peer educators and covering a broad curriculum of health-related topics, including nutrition, mental health, sexual and reproductive health, and violence. Peer education interventions are broadly defined as interventions in which adolescent or young adult facilitators seek to increase adolescents’ knowledge or influence their attitudes [9]. Interventions facilitated by peers may have several advantages over those delivered by adult providers. First, working with young laypersons could reduce costs and thereby facilitate scale-up [10–12]. Second, peer opinion strongly influences behaviours in adolescence, and working with peers to shape norms is now seen as essential to the success of adolescent health campaigns [13]. Third, through their social networks, peers may be able to reach marginalised adolescents who are not otherwise engaged in formal health or education programmes.

Peer education interventions have been incorporated into numerous national adolescent health strategies as well as non-governmental programmes [14–17]. Some have been tested in India [18]. The RKSK peer educator curriculum has never been evaluated. Although some RKSK activities have started, such as adolescent-friendly clinics and the recruitment of peer educators, many evidence gaps remained when the curriculum was launched. What would adolescents and their families want from such an intervention? Who should the peer educators be? What kinds of activities should they perform to keep adolescents engaged in meetings? What complementary community-based activities are required to succeed in improving adolescent health and development? To answer these questions, we conducted 18 months of formative research aimed at optimising the Indian government’s proposed community intervention with peer facilitators.

### Formative research to develop a community adolescent health intervention

We used the Medical Research Council framework for the development and evaluation of complex interventions to develop an intervention for adolescent health and development in rural Jharkhand, eastern India [19]. We built on the Indian government’s proposal for peer facilitators and adolescent groups, assuming that they would eventually be implemented in our study area. Our formative research included six main components:

1. A systematic review of the effects of peer-facilitated interventions on adolescent health in LMICs [20]
2. A review of Indian and local adolescent health initiatives
3. A baseline survey with data from 3324 adolescent girls aged 10–19 to explore health and development (including nutrition, education and psychosocial well-being) across 50 villages in Jharkhand [21]
4. A survey of community resources for adolescents (schools, youth clubs, sports teams and community health services)
5. Thirteen focus group discussions with adolescent girls and boys aged 10–19, and 15 interviews with adolescents and frontline health workers
6. A community-based workshop with adolescents, parents, teachers and local experts in adolescent health and child protection, and a workshop with ten experts in adolescent health in India, to triangulate findings and gather ideas for the design of specific components of the intervention

Our global systematic review found that peer-facilitated interventions could be beneficial across a range of areas of health, with the strongest evidence for mental health [20]. Our review of Indian national and local adolescent health programmes found that many existed but had been designed and implemented by multiple governmental and non-governmental agencies, resulting in a lack of coordination and overlap. In eastern India in particular, there was discrepancy between planned and actual coverage. Our baseline survey of 3324 girls aged 10–19 years in 50 villages in Jharkhand provided additional formative data [21]. We found substantial school drop-out after the age of 15: less than half of girls aged 15–19 were still in school. Girls left school because they were required for work at home or on the family farm or business. Around half of all girls were too short for their age (height-for-age z score less than − 2 SD), around 10% were too thin for their age (body mass index-for-age z score less than − 2 SD), and less than one-fourth had received minimum dietary diversity in the last 24 h. Violence, especially emotional violence, was common, particularly among younger girls. Around one in ten girls reported problems related to depression or anxiety. Girls’ most common self-reported health problems were high fever and menstrual problems. Only 30% of girls aged 15–19 years had heard of contraception.

Participants in qualitative discussions described numerous social, behavioural and environmental challenges to adolescent health. Some, including lack of access to health facilities, violence at home and in school, trafficking and lack of livelihood opportunities, affected both boys and girls. Others were more sex-specific: Alcohol use was more common among boys, whereas girls were exposed to sexual harassment (described as ‘eve-teasing’), early marriage, early childbearing, and lack of parental and financial support for secondary education. Among all participants, there was a consensus that any community intervention for adolescents should go beyond improving health and help adolescents gain education and vocational skills. Adolescents, parents and teachers indicated that information alone would not engage adolescents and that groups should also be fun and social. Local facilitators close in age to adolescent group members, with engaging personalities and an informal approach, could help make the groups more age-appropriate, enjoyable and empowering.

Informed by findings from the reviews, survey and qualitative study, we developed a community intervention to be tested in a trial, building on the government-supported peer facilitators. The intervention’s components and working theory of change are described below.

### Study aim

We aim to assess whether an intervention involving a community youth team facilitating participatory peer-led adolescent groups, youth leadership activities and livelihood promotion can improve school attendance, nutrition and mental health among adolescent girls in rural India.

## Methods

### Setting

The study is located in West Singhbhum, a largely rural district in the eastern state of Jharkhand. West Singhbhum has a population of 1,502,338 [22]. Sixty-seven percent of its population belong to Scheduled Tribes who tend to be socio-economically disadvantaged compared with other demographic groups [22]. One-third of the population are below the age of 15 [23]. Eighteen percent of adolescent girls aged 10–19 cannot read, and a further 30% read with difficulty [21]. More than one-fifth of girls aged 15–19 are married [21].

The study is led by Ekjut (www.ekjutindia.org), a civil society organisation, in collaboration with University College London and the London School of Hygiene and Tropical Medicine. Ekjut have been working in West Singhbhum since 2004 to improve the health of women and children in rural tribal communities.

### Trial design

We are undertaking a parallel-group, two-arm, superiority, cluster-randomised controlled trial with 1:1 allocation ratio and two cross-sectional surveys, one at baseline and one at endline. The main trial analysis will be a cross-sectional comparison of data from the endline survey, adjusted for baseline differences. We chose this approach in favour of a longitudinal design (i.e. only re-interviewing baseline participants at endline), as we anticipated that a large (potentially > 50%) proportion of older adolescents (15–19 years) exposed to the intervention would move out of the district or state for employment opportunities or marriage and thus would be lost to follow-up with a longitudinal study design. We also thought that matching participants between baseline and endline would be challenging. Maximising our chances of including older adolescents exposed to the intervention was important, and the cross-sectional design allowed us to interview older girls who had potentially been exposed to the intervention.

The trial includes 38 clusters. Each cluster is a purposively selected geographic area with a population of around 1000 people (between 723 and 1962), comprising a village and its associated hamlets. Clusters are separated by natural boundaries in order to minimise contamination between intervention and control arms.

### Randomisation

**Fig. 1:**
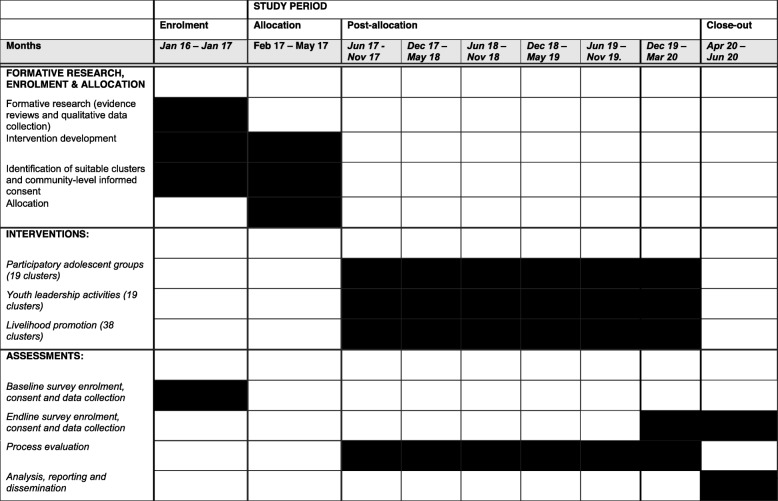
Trial design

We conducted the randomisation on the 22nd of February 2017, allocating 19 clusters to the intervention arm and 19 to the control arm. Clusters were stratified on the basis of five strata: (1) clusters with a secondary school and an adolescent club (clubs are community-based health and development groups for adolescents run by the Integrated Development Foundation), (2) clusters with a secondary school and no adolescent clubs, (3) clusters without a secondary school but with an adolescent club, (4) clusters without a secondary school and without an adolescent club, and (5) clusters having a population of more than 1500. Figure 1 describes the study profile. To ensure transparency of the randomisation process, we invited participants from the local community (village headmen, community health workers and a local government representative) to participate in the randomisation. We explained the intervention and the purpose of randomisation to the participants. Clusters were numbered and displayed on a wall. Identical balls were numbered 1 to 38, representing each of the 38 clusters. For each of the five strata, participants placed the numbered balls corresponding to the clusters in that strata into a local tombola device. They operated the machine and sequentially allocated each ball to the intervention and control arms. Allocation was not concealed to participants present during the randomisation.

### Trial participants and target population

All adolescent girls aged 10–19 years living in the 38 study clusters during the baseline and/or endline surveys are eligible to participate in study interviews. Girls who decline to be interviewed or who are living outside the study clusters are excluded. Data from our baseline survey suggest that around 15% of adolescent girls may be living outside the study clusters for reasons including studying away from home and being employed outside the village.

All adolescent boys and girls, aged 10–19 years, within the study area (whether living there or not) are eligible to participate in the intervention. Although, for financial and logistical reasons, our trial outcomes relate only to girls, we decided to include both boys and girls in the intervention because the intervention activities were relevant and potentially beneficial to boys and because some health-related problems, such as gender-based violence, early marriage and sexual and reproductive health, may be more effectively addressed by engaging with both boys and girls. Participation in intervention activities is voluntary, and adolescents are free to join or leave at any time.

### Intervention strategy

The intervention is a community youth team that delivers participatory adolescent groups, youth leadership activities and livelihood promotion. Additional file 1 describes the intervention’s full theory of change. Additional file 2 is a short video clip with Ekjut team members describing the intervention. Each cluster has a community youth team delivering parallel intervention activities. The team comprises a peer facilitator (*yuva saathi*, meaning “friend of youth”) aged 20–25 years, a youth leadership facilitator (one for six clusters), and a livelihood promoter (one for ten clusters). The second member of the community youth team is a youth leadership facilitator who delivers fun, confidence-building activities for adolescents every 2 months. Activities are open to all girls and boys in the community and include sports events such as football tournaments, archery and run-a-thons, as well as problem-solving sessions and nature walks. Both intervention and control clusters have livelihood promoters, who are adults recruited for their skills in farming and environmental management. Livelihood promotion activities aim to provide adolescents with practical skills which they can use in later life and that improve food security for families.

The intervention engages and supports frontline health care providers by inviting them to participate in adolescent group meetings and facilitating referrals of adolescents from the community youth teams to community health services. An advisory committee involving representatives from local governmental and non-governmental adolescent services also advises and supports the community youth teams on child protection issues and referral services. We describe the community youth team’s activities further below.

#### *Yuva saathis* and participatory adolescent groups

The main role of the *yuva saathi*s is to facilitate monthly participatory groups for adolescent girls and boys over a period of 36 months. Ekjut recruited 30 *yuva saathi*s (20 females and 10 males) in total. The recruitment process involved a general knowledge test and face-to-face interviews with an assessed role-play. *Yuva saathi*s were recruited on the basis of their ability to speak both Hindi and Ho (the most common local tribal language spoken in the study area), their performance on a general knowledge test, and their ability to demonstrate confidence and good communication skills during a face-to-face interview. We chose to recruit *yuva saathi*s aged 20–25 years because our formative research findings suggested that adolescents preferred facilitators who were slightly older than them, and parents, teachers and health workers felt that facilitators in this age group would have more confidence to facilitate meetings. *Yuva saathi*s are paid INR 5000 per month as an incentive and are trained by members of the Ekjut team. They are supervised by coordinators and supervisors who observe approximately 20% of group meetings and meet with *yuva saathi*s fortnightly to debrief, troubleshoot and plan future meetings. If *yuva saathi*s have any concerns about adolescents in their group, coordinators and supervisors help them organise referrals to health facilities or other services.

In each cluster, *yuva saathi*s facilitate meetings which are mainly held in community meeting spaces. The first five meetings aim to introduce adolescents to the intervention. Groups discuss social and economic influences on adolescents’ health, how to identify and involve vulnerable adolescents in the intervention, gender norms and their consequences, and the adolescents’ own needs and expectations. These initial meetings are open to all community members, including adolescent girls and boys, their parents, teachers and frontline health workers, and *yuva saathi*s actively mobilise community members to attend. All meetings involve participatory games and open discussion.

After the first five meetings, the groups work through four consecutive Participatory Learning and Action (PLA) cycles. We chose to use a PLA approach for these meetings because there was a consensus that building adolescents’ confidence and decision-making skills would be desirable, and Ekjut has substantial experience with this method. In earlier trials, PLA interventions enabled communities to improve the survival of newborn infants, increase the dietary diversity of pregnant women and young children, and reduce underweight in young children [24–27]. PLA is now an approach used by the India National Health Mission to conduct health-related meetings in the community and may be extended to peer facilitators of adolescent groups [28].

Each PLA cycle comprises five to seven meetings and has four distinct phases: (1) identifying problems affecting adolescents in the community (meeting 1), (2) identifying and deciding on strategies to address these problems (meetings 2–3), (3) implementing the strategies (meetings 4–6), and (4) evaluating the process (meeting 7). The same *yuva saathi* facilitates each meeting. There is a PLA cycle for each of the following four themes: education, nutrition, health and violence. The themes were selected on the basis of our formative research and reflect the broad dimensions of adolescent health and development as well as the RKSK curriculum. At the start of each cycle, adolescents are given a choice of meeting in single-sex or mixed groups. A discussion is also held with local governance bodies, frontline health workers and teachers to seek their ongoing consent for the meetings, because topics (e.g., mental health, violence or sexual and reproductive health) are considered sensitive. In the first phase of each PLA cycle, in order to stimulate discussion, *yuva saathi*s use picture cards showing problems that adolescents might face. Problems represented on the picture cards under each theme are shown in Table 1 and are standardised across groups. Groups then vote on three problems that they would like to address, and they select one or two for further discussion. Problems mentioned by adolescents that are not represented on the picture cards are written down on blank cards and included in the voting exercise. In the second phase of each cycle, *yuva saathi*s use stories based on prioritised problems to help groups examine the causes of problems they identified in the first phase. The stories prompt groups to consider causes at the family, community and societal levels. Groups decide which of these causes they would like to address, develop appropriate strategies, and identify ways to evaluate these strategies. In the third phase, groups implement their chosen strategies. During this phase, groups also participate in meetings to explore some of the problems that were not prioritised but are considered relevant in light of the formative research. At the start of each meeting in this phase, the group devotes around 15 min to discuss and review their strategies, challenges faced, and strategies for overcoming these challenges. In the final phase, groups review their strategies, any challenges they faced, and how these challenges were overcome. They also organise a community meeting at the end of each PLA cycle, during which groups share their experience and learning and seek support from the wider community. Strategies implemented in earlier cycles continue to be implemented throughout the intervention implementation period. A community meeting is held at the end of each of the four thematic PLA cycles, and an overall evaluation meeting to discuss all strategies implemented and the way forward is also held at the end of the intervention.

**Table 1:** Problems represented on picture cards used in Participatory Learning and Action cycles

Theme	Problems represented on the picture cards^a^
Education	Gender norms related to education
	School drop-out
	Lack of access to school-related entitlements
Nutrition	Anaemia
	Lack of access to nutrition-related entitlements
	Inadequate dietary intake and dietary diversity
	Intra-household food distribution
	Intra-household food insecurity
Health	Lack of menstrual hygiene and menstrual disorders
	Early marriage and adolescent pregnancy
	Alcohol and substance abuse
	Depression and anxiety
	Behavioural disorders
	Lack of access to health entitlements
Violence	Street harassment
	Physical and emotional violence
	Sexual harassment
	Not being able to voice their opinions

^a^Problems mentioned by adolescents that are not on this list will be written down on blank cards and included in the prioritisation exercise

Through the PLA cycles, we expect groups across the intervention arm to have devised and implemented a wide range of strategies to address key health and development problems affecting adolescents in their communities. The PLA cycles are expected to improve adolescents’ knowledge about health, education and nutrition (including services and entitlements), and gender equity. Through the meetings, adolescents are expected to gain confidence to share their needs and problems within the group, with their parents and peers, as well as to improve their problem-solving skills and ability to address issues related to their own health and development. Marginalised adolescents, especially those who are out of school, were identified during the five introductory meetings and specifically encouraged to join the groups. PLA cycles are also expected to have effects on adult members of the community, including increasing their knowledge about the health and development needs of adolescents, motivating them to support the adolescent groups and their strategies, and helping adults to recognise and appreciate adolescents as citizens of the community with their own rights and entitlements.

#### Youth leadership facilitators

Research from other settings in India suggests potential benefits of combining peer-facilitated interventions with complementary activities [18], and that developing adolescents’ self-efficacy and psychosocial resilience is a ‘missing piece’ in improving girls’ health [7]. Our formative research also indicated that adolescents would like opportunities to participate in cultural and sports activities in their communities. On this basis, the community youth team involves a youth leadership facilitator to deliver fun activities that build adolescents’ confidence and help to keep them engaged in the PLA cycle. These activities are also an opportunity to provide information related to adolescent health and development, and to reach out to the rest of the community. Activities are open to all girls and boys in the community and occur every 2 months. They run in parallel with the PLA cycle and include football tournaments and other sports activities, problem management sessions, cycling sessions and nature walks. Youth leadership facilitators are local adults recruited by Ekjut on the basis of their leadership skills, experience of working with young people, and understanding of the principles of PLA. Facilitators participate in monthly meetings with *yuva saathis*, coordinators and supervisors in order to coordinate and plan intervention activities.

#### Livelihood promoters

Through our formative research, adolescents and their parents informed us that they wanted opportunities to participate in livelihood training to develop practical skills related to farming and environmental management. We therefore engaged livelihood promoters, who are adults recruited for their skills and knowledge in farming practices and environmental management, to deliver a programme of livelihood promotion activities. Activities reflect the seasons, are selected in consultation with communities and include paddy cultivation, multi-cropping, compost-making and other organic farming techniques, tree planting, rainwater harvesting, and revival of farmers and save the forest groups (*van samitiy*). Activities will run approximately every 3 months in both intervention and control arms and are family-focused, involving both adolescents and their parents. The programme has three main aims: (1) to provide adolescents with practical skills that they can use in later life; (2) to improve food security for families, which will help to improve dietary diversity; and (3) to provide a common benefit to both intervention and control arms to help build support for the research across the trial arms.

### Trial timeline and status

**Fig. 2:**
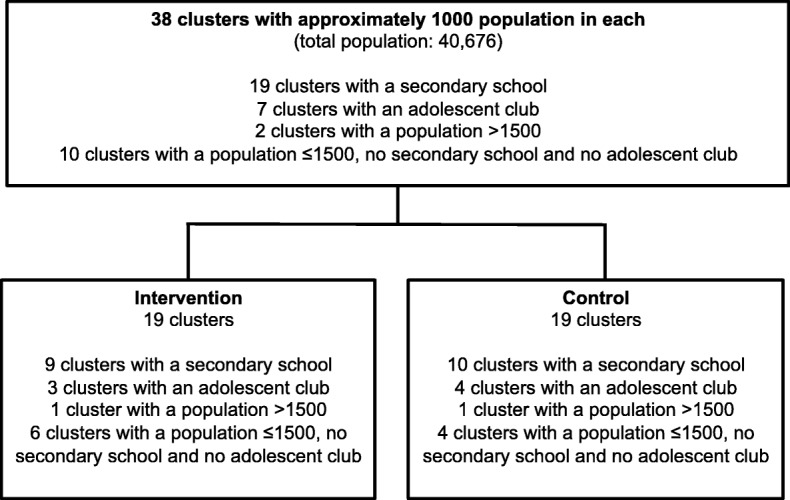
Study timeline

The baseline survey was conducted between June 2016 and January 2017. In April and May 2017, we recruited and trained the community youth teams. The intervention will be implemented over 36 months from June 2017 to May 2020. The endline survey will partially overlap with intervention implementation and will be conducted between December 2019 and April 2020. A time frame for the research activities is shown in Fig. 2.

### Research questions

We seek to test the effects of the intervention on three main outcomes: school attendance, dietary diversity and mental health. We chose these to represent the broad range of health and development issues affecting adolescent girls in India and because formative research indicated that these are important problems for girls in the study area.

#### Primary research questions

What is the effect of an intervention comprising participatory adolescent groups, youth leadership activities and livelihood promotion, delivered by a community youth team, on adolescent girls’ school attendance, dietary diversity and mental health?

#### Secondary research questions

Secondary research questions explore intermediate effects of the intervention on the pathway to impact on school attendance, dietary diversity and mental health. We ask, does the intervention:

- increase the uptake of school-related entitlements (e.g., cash, bicycles, books, mid-day meals for girls in upper primary, scholarships)?
- reduce the number of girls who were absent from school in the past 2 weeks?
- increase adolescent girls’ decision-making ability, self-efficacy and resilience?
- reduce the percentage of girls who drank alcohol in the past month?
- increase the percentage of girls with attitudes supportive of equitable gender norms related to education and domestic work?
- reduce exposure to emotional and physical violence and increase the number of girls who intervene to reduce emotional and physical violence against their peers?

### Study outcomes

The trial has three primary outcomes: school attendance, dietary diversity and mental health problems (Table 2). We will assess the percentage of girls attending school or college at the time of the endline survey. Information on school attendance will be self-reported, and we will cross-check answers in school registers for 10% of girls randomly selected from the sample. We will use the Food and Nutrition Technical Assistance tool to measure mean dietary diversity score (based on 24-h recall) [19]. For mental health problems, we will compare the mean score on the Brief Problem Monitor–Youth (BPM-Y) across trial arms [20]. The BPM-Y includes items on internalising (depression and anxiety), externalising (conduct disorder and oppositional defiant disorder) and attention problems. Secondary outcomes on the pathway to change for the primary outcomes are outlined in Table 2 and presented in the theory of change. Tertiary outcomes are also shown in the theory of change (Additional files 1 and 3) and relate to further hypothesised outcomes of the intervention that are not directly related to the primary outcomes. Additional file 3 also outlines how each outcome is measured and if baseline data are available.

**Table 2:** Trial outcomes

Primary outcomes	
1	Percentage of adolescent girls attending school or college
2	Mean dietary diversity score, based on 24-h recall
3	Mean score on the Brief Problem Monitor–Youth
Secondary outcomes	
1	Percentage of girls making decisions independently and with others about the food they eat, including how much they eat and what types of food they eat
2	Mean score on gender role attitudes index
3	Percentage of girls making decisions independently and with others about friends, spending money and purchases
4	Mean score on the Schwarzer General Self-Efficacy (GSE) Scale
5	Mean score on the Child and Youth Resilience Measure 11-item version (CYRM-B)
6	Percentage of girls who report experiencing emotional violence in the past 12 months
7	Percentage of girls who report experiencing physical violence in the past 12 months
8	Percentage of girls who report intervening to reduce emotional violence against their peers in the past 12 months
9	Percentage of girls who report intervening to reduce physical violence against their peers in the past 12 months
10	Percentage of girls who report being absent from school in the past 2 weeks
11	Percentage of girls accessing at least one school-related entitlement (cash, bicycles, books, midday meal scheme)
12	Percentage of girls who drank alcohol in the past month
Tertiary outcomes	
1	Percentage of girls who took at least four iron and folic acid supplements in the past month
2	Percentage of girls aged 15–19 and all married girls who have correct knowledge about the contraceptive pill, condoms and the IUD
3	Percentage of girls who use sanitary napkins or clean cloths during their period
4	Percentage of girls aged 15–19 and all married girls who know that abortion is legal
5	Percentage of girls who have received take home rations in the past month
6	Percentage of girls underweight (less than −2 SD median BMI for age and sex)
7	Percentage of girls stunted (less than −2 SD median height for age and sex)
8	Mean MUAC score

*BMI* body mass index, *IUD* intra-uterine device, *MUAC* Mid Upper Arm Circumference

### Sample size and power

The size of the study area, and hence the number of clusters, was chosen for logistical reasons. With a district-level crude birth rate of around 23 per 1000 population and accounting for child deaths, we expected to find an estimated 8800 adolescents aged 10–19 (4400 girls) in our proposed intervention area. We anticipated that in each cluster there would be ~115 adolescent girls aged 10–19 years.

A total of 3324 adolescent girls aged 10–19 years participated in the baseline survey: 82% of an estimated 4068 girls in the 38 clusters. The mean number of girls in each cluster was 87 (SD, 29.9). We aim to interview the same number of girls in the endline survey. With a mean cluster size of 87, a coefficient of variation of cluster sizes of 0.3 [29], and using intra-cluster correlation coefficients (ICCs) from our baseline data, the trial will have 80% power to detect a nine-percentage-point increase in the proportion of girls attending school or college (ICC, 0.03), from a baseline prevalence of 69% to 78%; a 0.9-point increase in mean dietary diversity score (ICC, 0.40), from a mean baseline score of 3.4 (SD, 1.4) to 4.3; and a 2.7-point decrease in BPM-Y score (ICC, 0.39) from a mean baseline score of 6.0 (SD, 4.3) to 3.4. The significance level is set at 0.05. We performed these calculations in Stata software (version 14; StataCorp, College Station, TX, USA). We anticipate that including baseline data in our analysis will lead to gains in power but do not attempt to quantify these here.

### Data collection, cleaning and storage

In this section, we describe data collection processes for the endline survey. These are identical to processes followed at baseline, which are described elsewhere [21]. Twelve monitors who are independent from the community youth teams will undertake data collection. In each of the study clusters, they will visit all households in order to identify eligible adolescent girls. In households with an eligible girl, monitors will explain the study to the girl and seek her consent. For girls under the age of 18, monitors will also seek consent from the girl’s caregiver. The monitor will then find a convenient time to conduct an interview with the girl in her home (e.g., outside school hours for school-going girls). If girls are not available when the monitor visits the household, the reason for unavailability will be recorded. Through these interviews, we will collect data relating to the primary and secondary outcomes, as well as data on the socio-economic status of the household and for the process evaluation. Data will be collected using mobile phones programmed using the CommCare data collection platform, which uses automated skip patterns and in-built checking and consistency logic to support data validation. Data will be downloaded from the server every 2 weeks. We will check the number of interviews completed and look for any problems. Every month, we will check the data for outliers, errors and missing data using automated do-files in Stata. The final dataset will be stored on a password-protected hard drive after removing individual identifying information.

### Blinding

Data collection during the endline survey will overlap with intervention activities by approximately 3 months. Monitors will not be given information about the allocation of clusters. However, through their monitoring activities in the villages, they may encounter some of the intervention activities or meet members of the community youth teams. Because of the participatory nature of the intervention, adolescents and the community youth teams cannot be blind to allocation. The researcher conducting the final trial analyses will be blind to allocation. Analyses for the primary outcomes will be repeated by an independent statistician also blind to allocation.

### Analysis plans

Primary analysis will employ an intention-to-treat approach, including all adolescent girls aged 10–19 living in the study area, regardless of their level of participation in the intervention activities. We have three primary outcomes. We think that success in improving at least one of these outcomes could help inform decisions about future scale-up. We will declare the trial a success if we find a significant (two-tailed *P* < 0.05) benefit for at least one outcome in conjunction with a collectively ‘positive signal’ for the other two outcomes. A ‘positive signal’ is defined as at least one of the two outcomes in the direction of benefit, neither outcome showing significant harm, and if for one outcome the direction of effect is towards benefit and for the other it is towards harm, then we require the (two-tailed test) *P* value for the former outcome to be smaller than that for the latter. The type I error rate corresponding to this definition of success varies according to the correlations between the three outcomes, which we expect to be positive. Under perfect positive correlations, the type I error rate is 5%. Under independence and the null hypothesis (no intervention effect on any outcome), the probability of observing success in conjunction with one, two or three significant benefits observed for individual outcomes is 3.38% (3 × 0.025 × 0.95^2^ × 0.5), 0.18% (3 × 0.025^2^ × 0.95) and 0.00% (0.025^3^), respectively. Consequently, the overall probability is 3.56%, and the type I error rate assuming a symmetric definition of overall trial failure (harm) is 7.12%. For primary and secondary outcomes, we will conduct cross-sectional analyses to compare differences between the control and intervention arms of the trial, using regression models with generalised estimating equations (GEEs) to adjust for clustering and model baseline and endline information together. We will use the exchangeable working correlation structure. To assess the effect of the intervention, our regression models will include an indicator of time (endline vs. baseline) and an indicator of intervention, coded 1 for participants measured at endline in the intervention arm and 0 otherwise. This analysis approach has been termed a ‘constrained baseline analysis’ [30]. Our baseline data show that dietary diversity is slightly positively skewed and BPM-Y scores are very positively skewed. We will model mean dietary diversity scores using linear regression. We will model BPM-Y scores using linear regression after transformation by the function log(1 + *x*), noting that our analytic approach (marginal model fitted by GEE) provides robustness by avoiding distributional assumptions. We will use a logistic regression model to test for a difference in the percentage of girls attending school between arms as a binary outcome. We will adjust for the same set of pre-specified prognostic factors (asset quintile, tribal status and age) and for strata for each outcome. We will additionally adjust for further socio-economic factors, should the data monitoring committee note important imbalances between arms among baseline participants. However, because there could be collinearity between such factors or between such factors and the pre-specified prognostic factors, it may be that not all can be included or that some may need to combined. The final model will be selected without reference to intervention effect (i.e., without seeing the impact of different potential models on the intervention effect estimate). We do not plan to conduct any sensitivity analysis for the effects of missing data, because the refusal rate for the endline survey is expected to be low (< 1%), in line with the baseline survey. We think that short-term cross-over between intervention and control areas is likely to be rare, because the main reason for moving is to attend boarding school, and there are few boarding schools in our study clusters. We will be able to identify participants who have crossed over through their exposure to the intervention and will provide an estimate of ‘contamination’, though we expect this to be less than 1% of participants.

We plan to carry out two sub-group analyses for the primary outcomes. The first will assess the effects of the intervention on the primary outcomes by age, categorising girls as younger (10–14 years) or older (15–19 years). The second will examine intervention effects on the primary outcomes by household wealth quintile. For sub-groups of age and wealth quintile, the intervention effects will be presented within each sub-group following the same approach as in the main analyses. Formal testing for a difference in intervention effect across sub-groups will be based on testing interaction terms. Tertiary outcomes that are present in the theory of change will be reported in the process evaluation as exploratory analyses.

We will convene a data monitoring committee in 2019 and 2020 to (1) examine the comparability of trial arms and potentially identify socio-economic variables that differ between arms to adjust for in the final analysis, (2) approve our data analysis plan and ensure we conduct the final analyses in accordance with this, and (3) provide recommendations on the interpretation of results and additional analyses.

The anonymised trial dataset with data on primary and secondary outcomes and code used in the main analyses will be made available as supplementary files with the main trial publication.

### Process evaluation

Through a process evaluation, we will assess intervention implementation (participation in the intervention and whether the intervention was delivered as intended), mechanisms of impact, and context (effect of context on implementation and outcomes) [31]. Process evaluation data will be collected using (1) semi-structured interviews and focus group discussions with adolescents and members of the community youth team in purposively selected clusters, (2) observation of intervention activities and interactions with adolescents and other community members, (3) forms to document attendance at intervention activities and to capture problems and strategies identified by the participatory adolescent groups, and (4) data collection through the endline survey to measure participation of adolescents in intervention activities. Some of the information will be collected quarterly/monthly, whereas other information will be collected towards the end of the intervention. Information that will be collected on an ongoing basis includes socio-demographic profiles of participants attending intervention activities, prioritised problems and strategies, progress on implementing strategies, spontaneously reported adverse events, training feedback based on the meeting contents, and feedback from participants during meetings. Group discussions and semi-structured interviews with adolescents, parents and frontline workers will be conducted at the end of the intervention. We do not have financial resources to conduct a full economic evaluation but will report intervention costs from a provider perspective to guide future decisions on implementation.

### Ethical considerations

In May 2016, we obtained ethical approval for the study from an independent ethics committee in Jharkhand convened by Ekjut and from the University College London Ethics Committee. The study presents several ethical considerations.

#### Need for cluster-level consent from village leaders and individual-level consent from girls and their parents/caregivers

Ekjut have previously worked in the study area and are familiar with the village leaders and communities. We sought consent for each village’s participation from the local village governance institutions (Panchayat and headmen) and opinion leaders after explaining the study’s purpose and processes. Adolescent girls aged 18 or above were asked to provide informed consent for themselves in the baseline survey, and this will be repeated at endline. For girls aged 10–17, we will first seek informed consent from their parents/caregivers. We will not collect data from any girls if they have not themselves provided assent, regardless of whether their caregivers have.

#### Ensuring confidentiality

As during the baseline, monitors will conduct interviews for the endline survey in or around girls’ homes in a place of the girl’s choosing, ideally out of earshot but within sight of family and neighbours. This will protect confidentiality and ensure that the girl feels comfortable and safe during the interview. Data will be collected on mobile phones, stored on password-protected computers and backed up every 2 weeks. Names of participants will be removed from the final datasets.

#### Identification of severely anaemic, severely acutely malnourished, or mentally distressed girls and girls exposed to severe violence

As in the baseline survey, all girls showing symptoms of severe anaemia and those who are severely acutely malnourished, in both intervention and control arms in the endline, will be referred to the nearest health facility. Girls with severe mental health problems or who report experiencing sexual or severe physical violence will be visited by a trained counsellor from Ekjut who will review their case and refer as appropriate to a local health facility or other relevant service (e.g., adolescent-friendly clinics under RKSK). All girls participating in the study will be provided with the contact details of a person in Ekjut.

#### Benefits to control areas

If the intervention is successful, we will apply for funding to scale it up in the control arm. During the trial, livelihood promoters will work in both intervention and control clusters to offer a range of activities related to farming practices and environmental management. This was chosen to offer a minimum common benefit to all trial areas to help build support for the research across trial arms.

#### Increased demand on local health services

Through the intervention, adolescents will be encouraged to seek help from community health services for various health care needs, including family planning, symptoms of anaemia and mental health problems. We will link the community youth team to the health workers by inviting them to attend participatory adolescent group meetings and supporting referrals of adolescents to community health facilities.

### Intervention generalisability, sustainability and scalability

We expect our results will be generalisable to other areas of rural India with a high proportion of Scheduled Tribes, such as Odisha, Chhattisgarh, Madhya Pradesh and Rajasthan.

The Government of India has committed to providing peer education through community groups across India through the RKSK programme. Our intervention model is potentially scalable through this programme. Through the study, we will explore how training and supervision of peer facilitators could be sustained by frontline health workers such as *Sahiya*s (accredited social health activist), *Sevikas* (Anganwadi worker) and auxiliary nurse midwives. We will also explore the feasibility of involving existing education and health personnel, such as teachers and health workers, in the delivery of the youth leadership and livelihood promotion activities.

### Dissemination

At the beginning of this study, we convened an expert group of representatives from the state government and from local and international non-governmental organisations working on adolescent health and development to participate in the design of the intervention. This helped to ensure the intervention is compatible with existing health and education systems, builds on the experience of local stakeholders, and is potentially scalable. We will re-engage this expert group at the end of the trial in order to disseminate results among those involved in adolescent health policy and programming. To disseminate our findings in the study area, we will hold community-level meetings, drawing on previously tested dissemination techniques (e.g., ‘traffic light signs’ to show which adolescent health outcomes have improved during the trial and which have not). We will also share results with the adolescents who participated in our intervention during the trial.

## Discussion

We have developed a holistic, participatory intervention to mobilise rural communities to take action for adolescent health and development, and we are evaluating it through a cluster-randomised controlled trial. To the best of our knowledge, this is the first trial in India of an integrated community intervention to improve adolescent health and development in tribal communities. Through the process evaluation, we aim to understand to what extent girls from these communities and other marginalised groups are able to participate in the intervention and how it achieves any effects detected. We will also try to understand factors that could facilitate or inhibit scale-up of the intervention in other tribal areas of India.

Several trials in India have evaluated community interventions for adolescents, mainly in school settings. A school-based intervention for girls aged 11–14 in Uttar Pradesh involving peer and parental support and sessions on compassion, well-being and self-efficacy improved nutrition, hygiene and reproductive health behaviours [32]. A psychosocial resilience curriculum for girls (mean age, 12 years) in Bihar improved gender-equitable attitudes; health knowledge and behaviours; and resilience, self-efficacy, social-emotional assets and well-being [7, 33]. In Goa, a pilot trial evaluated a multi-component intervention for boys and girls aged 16–24 in rural and urban communities [18]. The intervention reduced self-reported violence perpetration and probable depression, and it improved knowledge and attitudes about reproductive and sexual health in both rural and urban settings. A trial of a multi-level, structural and norms-based intervention in Karnataka found no evidence of a reduction in school drop-out and child marriage among girls aged 12–13 [34]. The whole-school SEHER (Strengthening Evidence base on scHool-based intErventions for pRomoting adolescent health programme) intervention delivered by lay counsellors in Bihar improved school climate and health-related outcomes among secondary school students (including depression, bullying and violence victimisation) [35].

Our study has several strengths. The intervention targets adolescents across the whole span of adolescence (10–19 years) and includes in- and out-of-school and married and unmarried adolescents. Involving boys and girls in the intervention activities will enable us to tackle issues related to gender norms and hopefully to provide benefits for both genders. The intervention builds on the government’s existing national adolescent health community strategy (RKSK). It has also been informed by an extensive mixed-methods formative study. The study also has limitations. Our primary outcomes are self-reported. Girls in intervention areas may be more likely to over-report mental health problems, minimum dietary diversity and school attendance due to awareness-raising intervention activities on these issues. We seek to address this in several ways. First, the data collection team is separate from the intervention team, reducing the extent to which participants relate the two, thus reducing potential social desirability bias. Second, interviewers are experienced and rigorously trained in rapport-building. This will help participants feel comfortable and able to answer questions truthfully. Third, for the school attendance outcome, we will cross-check a random sample of answers against school registers. Fourth, there may be migration of adolescents into and out of the study clusters. We will ask about length of residence in the cluster at endline to check for any systematic bias in in-migration between the intervention and control arms, though this is unlikely. Finally, because intervention activities are open to any adolescent, we cannot rule out the possibility that adolescents in control areas will participate. However, clusters were purposively selected so that natural boundaries (e.g., roads, rivers, forestry) between clusters reduce the likelihood of this.

Our findings will help to inform the implementation of RKSK and will contribute to the evidence base for community interventions to improve adolescent health and development in other settings.

### Trial status

The trial is ongoing. The endline survey will take place between February and April 2020.

## Supplementary information

**Additional file 1.** Intervention theory of change.

**Additional file 2.** Short video clip with the research team describing the intervention.

**Additional file 3.** Trial outcomes and detailed questionnaire items.

**Additional file 4.** SPIRIT 2013 checklist.

## Data Availability

The Standard Protocol Items: Recommendations for Interventional Trials (SPIRIT) checklist has been completed and made available (Additional file 4).

## Abbreviations

BMI: Body mass index
BPM-Y: Brief Problem Monitor–Youth
GEE: Generalised estimating equation
ICC: Intra-cluster correlation coefficient
IUD: Intra-uterine device
JIAH: Jharkhand Initiative for Adolescent Health
LMICs: Low- and middle-income countries
PLA: Participatory Learning and Action
RKSK: *Rashtriya Kishor Swasthya Karyakram*
