# Supplementary material for: Community youth teams facilitating participatory adolescent groups, youth leadership activities and livelihood promotion to improve school attendance, dietary diversity and mental health among adolescent girls in rural eastern India: protocol for a cluster-randomised controlled trial
Source: Trials. 2020 Jan 8;21:52. doi: 10.1186/s13063-019-3984-1 (PMC6950906; doi:10.1186/s13063-019-3984-1)
Supplement: Supplementary file 1 — Additional file 1. Intervention theory of change. [file 13063_2019_3984_MOESM1_ESM.pdf]

Resources

Community Youth Team

Yuva saathi ("Friends of Youth", peer facilitator, intervention clusters only)
- 30 yuva saathis aged 20-25 recruited from same village
- Able to speak Hindi and Ho
- Facilitate participatory learning and action (PLA) groups
- Liaise with PLA coordinators and supervisors regarding adolescent referrals
- Participate every 15 days in review meetings and training on PLA cycle content

Youth Leadership Facilitators (intervention clusters only)
- Facilitate youth leadership activities
- One per 6-7 clusters

Livelihood Promoters (intervention and control)
- One per 9-10 clusters to facilitate livelihood promotion activities

Support personnel

PLA Coordinators and Supervisors
- One coordinator and 1 supervisor per 15 yuva saathis
- Support yuva saathis to conduct PLA meetings
- Meet yuva saathis fortnightly to debrief, troubleshoot and rehearse future meetings
- Help to organise adolescent referrals to health and child protection services
- Participate in PLA training and observe ~20% PLA meetings

Health Care Providers
- Respond to referral requests and refer to primary/secondary facilities or other organisations as appropriate
- Participate in PLA meetings

Advisory Committee
- Comprising key stakeholders from local governmental and non-governmental adolescent organisations
- Advise PLA coordinators and supervisors and link with appropriate sectors

Community activities

Participatory adolescent groups , youth leadership training and livelihood promotion for adolescent girls and boys
(All in-school and out-of-school boys and girls aged 10-19 years are encouraged to participate)

Participatory adolescent groups
- One meeting per group per month
- During meetings yuva saathis share information through visual aids, games and stories
- For the first five meetings groups are mixed sex and involve parents and teachers; for subsequent meetings groups are single sex
- Groups work through 4 PLA cycles on education, health, nutrition and violence
- PLA cycles involve problem identification, deciding on strategies to address problems, implementing strategies and evaluating the process

Youth leadership training
- One youth leadership meeting every two months
- Involves cultural and sports activities to build self efficacy, communication skills, decision-making skills and problem-solving skills, and to promote adolescent health in the community

Livelihood promotion
- Activities every 3 months for adolescents and their parents
- Activities include farming, developing nutrition gardens and environmental management

Early effects (girls, boys, parents, teachers and health workers)

Adolescents (girls and boys)

Learning
- Adolescents are more informed about diet, menstruation and menstrual hygiene, mental health, and ways to promote emotional wellbeing
- Adolescents understand the importance of gender equity and how it relates to education, domestic work, eve-teasing and marriage
- Adolescents are more informed about health services
- Adolescents have increased knowledge about school and nutrition entitlements

Motivation
- Marginalised adolescents feel included in groups
- Adolescents are able to prioritise their own health, nutrition and development needs

Action
- Adolescents build and strengthen their social networks
- Adolescents communicate about their needs and problems with parents and peers
- Adolescents build self efficacy, communication skills, decision-making skills and problem-solving skills

Parents, teachers and health workers

Learning
- Parents, teachers and health workers have a better understanding about adolescent diet, menstruation and menstrual hygiene, mental health, and ways to promote emotional wellbeing
- Parents, teachers and health workers understand the importance of gender equity and how it relates to education, domestic work, eve-teasing and marriage
- Parents and teachers learn that physical violence adversely affects adolescent development and wellbeing
- Parents learn about the availability of school and nutrition entitlements for adolescents and how to access them

Motivation
- Parents, teachers and health workers are supportive of adolescent group meetings and strategies
- Parents, teachers and health workers recognise adolescents as citizens with their own rights and entitlements

Action
- Parents and teachers find ways to communicate with adolescents about their needs and problems, and avoid resorting to violence
- Parents make fewer demands on adolescent girls for household or farming work
- Parents provide girls and boys with equal opportunities related to education, marriage and nutrition

Later effects (girls only) *(*= secondary outcomes, # = tertiary outcomes)*

Health and nutrition
* Girls do not drink alcohol
* Girls make decisions independently and with others about the food they eat including how much they eat and what types of food they eat
# Girls take at least four iron and folic acid supplements each month
# Girls (15-19 and married girls) have correct knowledge of contraceptives
# Girls use sanitary napkins or clean cloths during their period
# Girls (15-19 and married girls) know abortion is legal
# Girls have increased access to take home rations
# Girls are not underweight or stunted, and have healthy MUAC scores

Gender norms and equity
* Girls make decisions independently and with others about friends, spending money and purchases
* Girls have attitudes supportive of gender equity in relation to education, relationships and domestic work

Mental health and wellbeing
* Girls have increased self efficacy and resilience
* Girls experience less emotional and physical violence in the past 12 months
* Girls intervene to reduce emotional and physical violence against their peers

Education
* Girls access school-related entitlements (cash, bicycles, books, midday meal scheme)
* Fewer girls are absent from school in the past two weeks

Primary outcomes (girls only)

Increased number of adolescent girls with minimum dietary diversity in the previous 24 hours
Reduced number of adolescent girls with mental health problems
Increased number of adolescent girls attending school or college
