# Supplementary material for: Community youth teams facilitating participatory adolescent groups, youth leadership activities and livelihood promotion to improve school attendance, dietary diversity and mental health among adolescent girls in rural eastern India: protocol for a cluster-randomised controlled trial
Source: Trials. 2020 Jan 8;21:52. doi: 10.1186/s13063-019-3984-1 (PMC6950906; doi:10.1186/s13063-019-3984-1)
Supplement: Supplementary file 3 — Additional file 3. Trial outcomes and detailed questionnaire items. [file 13063_2019_3984_MOESM3_ESM.docx]

**Additional file 3:** Trial outcomes and detailed questionnaire items

| **Primary outcomes** | | **Source of data** | **Baseline data available?** | **Type of outcome** | **Details** |
| --- | --- | --- | --- | --- | --- |
| 1 | Mean dietary diversity score, based on 24h recall | Endline survey | Yes | Continuous outcome | We will calculate a dietary diversity score based on 10 food groups:   1. Grains and tubers: roti, rice, peetha, other goods made from grains, mudhi/Food made from roots 2. Pulses: soya bean, other beans, peas, lentils 3. Nuts and seeds: Sunflower seeds, dori, mahua, kudrum, tisi, ramtia, linseed, sesame, chahar, mustard 4. Dairy: Cheese, yoghurt or other milk products 5. Meat, poultry and fish: liver, kidney, heart or other organ meat, chicken, duck or other birds, pork, lamb or goat, fresh fish, dried fish, shellfish or seafood, grubs, snails or insects 6. Egg 7. Dark green leafy vegetables 8. Other vitamin A-rich fruits and vegetables: pumpkin, carrot, sweet potato (orange/yellow inside)/Ripe mango, ripe papaya, ripe jackfruit 9. Other vegetables 10. Other fruits/Unripe mango, unripe papaya, unripe jackfruit |
| 2 | Mean score on the Brief Problem Monitor - Youth | Endline survey | BPC data only | Continuous outcome | Brief Problem Monitor (BPM)-Youth* questions, with possible edits following validation:   - I act too young for my age - I argue a lot - I fail to finish things I start - I have trouble concentrating or paying attention - I have trouble sitting still - I destroy things belonging to others - I disobey my parents - I disobey at school - I feel worthless or inferior - I act without stopping to think - I am too fearful or anxious - I feel too guilty - I am self-conscious or easily embarrassed - I am inattentive or easily distracted - I am stubborn - I have a hot temper - I threaten to hurt people - I am unhappy, sad, or depressed - I worry a lot |
| 3 | % of adolescent girls attending school or college | Endline survey | Yes | Dichotomous outcome | - Do you currently attend a school or college? |
| **Secondary outcomes** | |  |  |  |  |
| 1 | % of girls making decisions independently and with others about the food they eat including how much they eat and what types of food they eat | Endline survey | Yes | Dichotomous outcome | - Who usually makes decisions about the food that you eat, including how much you eat and what types of food you eat? (Read options and select more than one option if appropriate) |
| 2 | Mean score on gender role attitudes index | Endline survey | Yes | Continuous outcome | Gender role index:   - Do you think that educating boys is more important than educating girls? - Do you think that girls are usually as good as boys in studies? - Do you think that boys should do as much domestic work as girls? - Do you think that girls who are teased deserve it if they are dressed provocatively? - Do you think that a woman should obtain her husband’s permission for most of the things? - Do you think that girls should be allowed to decide when they want to marry? - Do you think that the husband alone/mainly should decide how household money is to be spent? |
| 3 | % of girls making decisions independently and with others about friends, spending money and purchases | Endline survey | Yes | Dichotomous outcome | - Who mainly takes the decision about who your friends would be? - Who mainly takes the decision about how to spend your money? - Who mainly takes the decision about what you buy? |
| 4 | Mean score on the Schwarzer General Self-Efficacy (GSE) Scale | Endline survey | No | Continuous outcome | GSE Scale:   - I can always manage to solve difficult problems if I try hard enough. - If someone opposes me, I can find the means and ways to get what I want. - It is easy for me to stick to my aims and accomplish my goals. - I am confident that I could deal efficiently with unexpected events. - Thanks to my resourcefulness, I know how to handle unforeseen situations. - I can solve most problems if I invest the necessary effort. - I can remain calm when facing difficulties because I can rely on my coping abilities. - When I am confronted with a problem, I can usually find several solutions. - If I am in trouble, I can usually think of a solution. - I can usually handle whatever comes my way. |
| 5 | Mean score on the Child and Youth Resilience Measure 11-item version (CYRM-B) | Endline survey | No | Continuous outcome | - I cooperate with people around me - Getting an education is important to me - I know how to behave in different social situations - People like to spend time with me - I feel supported by my friends - I feel that I belong / belonged at my school - My friends stand by me in difficult times - I am treated fairly in my community - I have opportunities to show others that I am becoming an adult and can act responsibly - I have opportunities to develop skills that will be useful later in life (like job skills and skills to care for others) |
| 6 | % of girls who report experiencing emotional violence in the past 12 months | Endline survey | Yes | Dichotomous outcome | Questions relating to emotional violence   - Has anyone ever: Cursed or insulted you, called you rude or hurtful names? - Has anyone ever: Humiliated or belittled you in front of other people, or embarrassed you? - Has anyone ever: Done things to scare or intimidate you on purpose, or threatened to hurt someone you care about ? - Has anyone ever: Forced you to stay inside or outside - Have you been made to work/look after siblings when you wanted to go to school? |
| 7 | % of girls who report experiencing physical violence in the past 12 months | Endline | Yes | Dichotomous outcome | Questions relating to physical violence   - Has anyone ever: Twisted your arm or any other body part, slapped you, pushed you, pulled your hair or thrown something at you? - Has anyone ever: Threatened to use or actually used a gun, knife or other weapon against you? - Has anyone ever: Choked you on purpose, dragged or beaten you up, tied you up with a rope or belt? - Has anyone ever: Punched you, kicked you, hit you with a closed fist or hit you with an object, such as a stick or a cane, or whipped you? - Has anyone ever: Severely beaten you up, cut you with a sharp object or burnt you purposefully? |
| 8 | % of girls who report intervening to reduce emotional violence against their peers in the past 12 months | Endline survey | No | Dichotomous outcome |  |
| 9 | % of girls who report intervening to reduce physical violence against their peers in the past 12 months | Endline survey | No | Dichotomous outcome |  |
| 10 | % of girls who report being absent from school in the past two weeks | Endline survey | Yes | Dichotomous outcome | - In the past two weeks did you have to miss any days of school/college ? (not including holidays or weekends) |
| 11 | % of girls accessing at least one school-related entitlement (cash, bicycles, books, midday meal scheme) | Endline survey | No | Dichotomous outcome |  |
| 12 | % of girls who drank alcohol in the past month | Endline survey | Yes | Dichotomous outcome | - Have you ever had any alcohol, other than a few sips? - During the last 1 month how often did you drink more than a few sips? |
| **Tertiary outcomes** | |  |  |  |  |
| 1 | % of girls who took at least four iron and folic acid supplements in the past month | Endline survey | Yes | Dichotomous outcome | In the past 1 month, have you received any iron and folic acid tablets?  How many iron and folic acid tablets did you consume in the past month? |
| 2 | % of girls aged 15-19 and all married girls who have correct knowledge about the contraceptive pill, condoms and the IUD | Endline survey | Yes | Dichotomous outcome | - How often should a woman take oral pills? - One condom can be used for how many acts of sexual intercourse? - Where is the IUD placed? |
| 3 | % of girls who use sanitary napkins or clean cloths during their period | Endline survey | Yes | Dichotomous outcome | Girls can use different methods for protection during their menstrual period to prevent bloodstains from becoming apparent. What do you mainly use for protection, if anything?   - Any cloth - Locally prepared napkins - Sanitary napkins - Other method - Nothing |
| 4 | % of girls aged 15-19 and all married girls who know that abortion is legal | Endline survey | Yes | Dichotomous outcome | If any woman has an unwanted pregnancy and wants to terminate it, is it legal for her to abort it? |
| 5 | % of girls who have received take home rations in the past month | Endline survey | Yes | Dichotomous outcome | In the past 1 month did you receive any Take Home Rations (THR) that were for you? |
| 6 | % of girls underweight (<-2SD median BMI for age and  sex) | Endline survey | Yes | Dichotomous outcome |  |
| 7 | % of girls stunted (<-2SD median height for age and  sex) | Endline survey | Yes | Dichotomous outcome |  |
| 8 | Mean MUAC score | Endline survey | Yes | Continuous outcome |  |
